# Nitric Oxide-Sensitive Guanylyl Cyclase Is Differentially Regulated by Nuclear and Non-Nuclear Estrogen Pathways in Anterior Pituitary Gland

**DOI:** 10.1371/journal.pone.0029402

**Published:** 2011-12-28

**Authors:** Jimena P. Cabilla, Silvana I. Nudler, Sonia A. Ronchetti, Fernanda A. Quinteros, Mercedes Lasaga, Beatriz H. Duvilanski

**Affiliations:** 1 Departamento de Química Biológica, Instituto de Química y Físico-química Biológicas, Facultad de Farmacia y Bioquímica, Universidad de Buenos Aires, Buenos Aires, Argentina; 2 Instituto de Investigaciones en Reproducción, Facultad de Medicina, Universidad de Buenos Aires, Buenos Aires, Argentina; University of Nebraska Medical Center, United States of America

## Abstract

17β-estradiol (E2) regulates hormonal release as well as proliferation and cell death in the pituitary. The main nitric oxide receptor, nitric oxide sensitive- or soluble guanylyl cyclase (sGC), is a heterodimer composed of two subunits, α and β, that catalyses cGMP formation. α1β1 is the most abundant and widely expressed heterodimer, showing the greater activity. Previously we have shown that E2 decreased sGC activity but exerts opposite effects on sGC subunits increasing α1 and decreasing β1 mRNA and protein levels. In the present work we investigate the mechanisms by which E2 differentially regulates sGC subunits' expression on rat anterior pituitary gland. Experiments were performed on primary cultures of anterior pituitary cells from adult female Wistar rats at random stages of estrous cycle. After 6 h of E2 treatment, α1 mRNA and protein expression is increased while β1 levels are down-regulated. E2 effects on sGC expression are partially dependent on *de novo* transcription while *de novo* translation is fully required. E2 treatment decreased HuR mRNA stabilization factor and increased AUF1 p37 mRNA destabilization factor. E2-elicited β1 mRNA decrease correlates with a mRNA destabilization environment in the anterior pituitary gland. On the other hand, after 6 h of treatment, E2-BSA (1 nM) and E2-dendrimer conjugate (EDC, 1 nM) were unable to modify α1 or β1 mRNA levels, showing that nuclear receptor is involved in E2 actions. However, at earlier times (3 h), 1 nM EDC causes a transient decrease of α1 in a PI3k-dependent fashion. Our results show for the first time that E2 is able to exert opposite actions in the anterior pituitary gland, depending on the activation of classical or non-classical pathways. Thus, E2 can also modify sGC expression through membrane-initiated signals bringing to light a new point of regulation in NO/sGC pathway.

## Introduction

Nitric oxide sensitive- or soluble guanylyl cyclase (sGC), the main intracellular receptor of nitric oxide is comprised of two subunits, α and β, of which several isoforms (α1, α2, α2i, β1 and β2) have been described. α1β1 is the most abundant and widely expressed heterodimer, showing the greater activity [1].

The major female hormone, 17β-estradiol (E2), is a key regulator of pituitary physiology involved in hormonal release as well as proliferation and cell death in anterior pituitary gland [2]–[4].

Previous studies from our laboratory show that acute E2 treatment exerts an inhibitory effect on sGC activity by down-regulating sGC β1 subunit in anterior pituitary gland. However, this treatment increases sGC α1 expression from both, immature and adult rats [5], [6]. The E2 effects on anterior pituitary sGC were observed not only after *in vivo* and *in vitro* treatment but also during estrous cycle. These observations support a direct effect of E2 on sGC regulation and a differential and independent regulation on both subunits. Previous evidence [7]–[9] further sustains that under certain conditions, α1 and β1 can be independently expressed.

E2 signaling pathways comprise classical and non-classical actions. Classical actions are mediated by nuclear E2 receptor (ER) and include both *de novo* transcriptional and translational events. E2 non-classical actions are mediated by non-nuclear ER and include the activation of signaling pathways that finally can also trigger transcription of certain genes [10], [11]. In many cells, around 5–10% of total ER is found at the plasma membrane, including both α and β ER subtypes depending on cell type [11].

E2 can also regulate many genes post-transcriptionally by affecting mRNA stability. Certain mRNAs have highly conserved sequences, adenine-uracil rich elements (AREs), present on untranslated 3′ end. AREs are involved in rapid mRNA degradation and are binding targets of several proteins. They constitute an important regulatory element involved in the control of genetic expression in vertebrates. Human antigen-R RNA binding protein (HuR) is ubiquitously expressed and belongs to embryonic lethal abnormal vision (ELAV) family proteins. HuR binds to AREs [12] and protects mRNA body from degradation. HuR is of major relevance since it can stabilize inducible nitric oxide synthase mRNA [13] and its expression seems to be directly related to ARE-containing mRNAs stability [14]–[17]. sGC α1 and β1 mRNAs include ARE sequences and both are able to bind HuR [18]. AREs elements are also a target of other factors such as heterogeneous nuclear ribonucleoprotein D (hnRNP D) also known as A+U-rich binding factor 1 (AUF1). This protein through competition with HuR regulates target mRNAs half-life and degradation [19]. The family of AUF1 proteins appears to be able to confer either stability or instability to target mRNAs, an effect being cell type and AUF1 isoform-dependent [20]. It has been previously shown that AUF1 expression is up-regulated by E2 in uterus [21], [22] and thus, can regulate the half-life of specific mRNAs [23]. Besides, it has been demonstrated that AUF1 binds sGC α2 mRNA in brain, which in turn decreases its half-life [24].

Taking into account this background we investigate whether E2 effect on sGC subunits is mediated through nuclear ER and/or non-nuclear ER and its mechanisms of action in anterior pituitary gland from adult female rats. Our results show a dual effect of E2 on sGC subunits expression depending on the activated ER pathway. E2 acting through non-nuclear ER, in a PI3K-dependent way, decreased α1 expression while acting through nuclear ER up-regulated α1 levels. On the other hand, sGC β1 subunit expression was also affected by E2 but apparently only through nuclear ER pathway. E2 actions on sGC mRNA levels through nuclear ER were dependent on *de novo* transcription and *de novo* translation, promoting an AREs-containing mRNAs destabilization environment. Altogether, our results provide the first evidence that E2 triggers opposite effects on sGC subunits expression through classical and non-classical pathways in anterior pituitary gland.

## Results

### E2 affects sGC subunits expression through both, nuclear and non-nuclear ER in an opposite way

**Figure 1:**
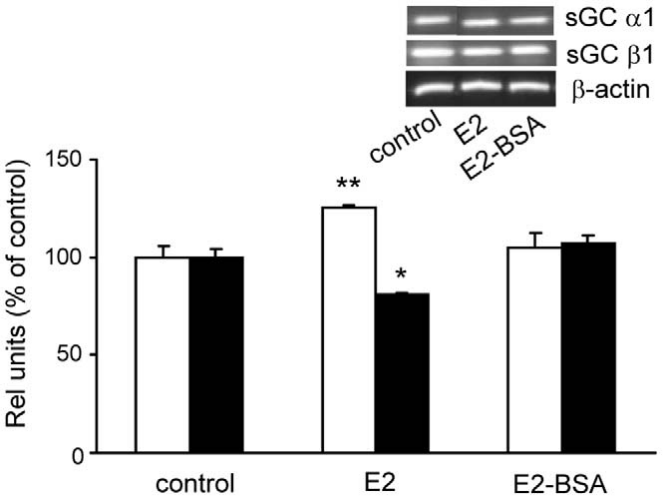
E2 actions on sGC α1 and β1 mRNAs were mediated by nuclear ER after 6 h of incubation. Pituitary cells in culture were incubated with vehicle (control) or 1 nM E2 or with 1 nM E2-conjugated to BSA (E2-BSA) unable to trespass cell membrane during 6 h. sGC α1 and β1 mRNAs were evaluated by PCR. *Top*, a representative PCR. *Bottom,* average densitometric values. Bars represent mean ± SE of relative units, corresponding to α1 (open bars) and β1 (black bars) densitometric values normalized to β-actin, and are expressed as percent of the control (n = 3). ANOVA followed by Tukey's test, *P<0.05, **P<0.01 *vs*. respective controls.

**Figure 2:**
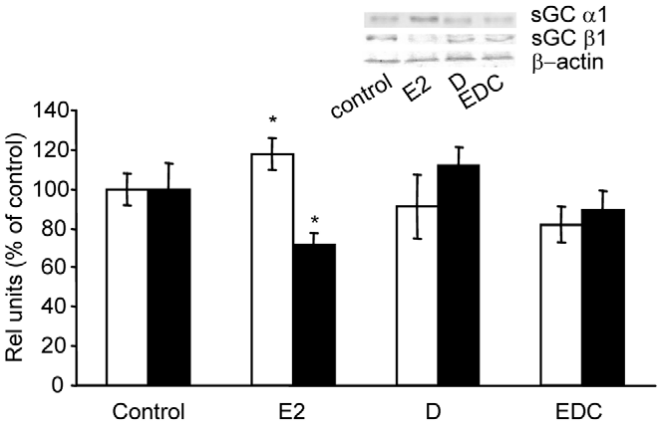
E2 actions on sGC α1 and β1 proteins were mediated by nuclear ER after 6 h of incubation. Pituitary cells in culture were incubated for 6 h with vehicle (control) 1 nM E2 or with 1 nM estrogen dendrimer conjugate (EDC) unable to trespass cell membrane, or with 1 nM dendrimer alone (D). sGC α1 and β1 proteins were evaluated by western blot. *Top*, a representative western blot. *Bottom,* average densitometric values. Bars represent mean ± SE of relative units corresponding to α1 (open bars) and β1 (black bars) densitometric values normalized to β-actin, and are expressed as percent of the control (n = 3). ANOVA followed by Tukey's test, *P<0.05 *vs*. respective controls.

Previously we have shown that E2 effects on sGC subunits occur specifically through ER [5]. To investigate which kind of ER is involved in such effect, anterior pituitary cell cultures were incubated with membrane-impermeable E2 compounds: bovine serum albumin-conjugated E2 (E2-BSA) and estrogen dendrimer conjugate (EDC) [25] using the free steroid (E2) as control. First we studied the effect of E2-BSA on α1 and β1 mRNA levels after 6 h of treatment, the same time previously assayed [6]. Our results show that 1 nM E2-BSA was unable to reproduce 1 nM free E2 effects on sGC subunits mRNA expression at the same concentration (**Figure 1**). At the same time period and similarly to E2-BSA effects, EDC could not reproduce E2 effects and α1 and β1 mRNA levels remained similar to control values (data not shown). EDC neither modified α1 or β1 protein expression (**Figure 2**) which further confirms that 6 h E2 treatment affects sGC subunits expression mainly through nuclear ER.

**Figure 3:**
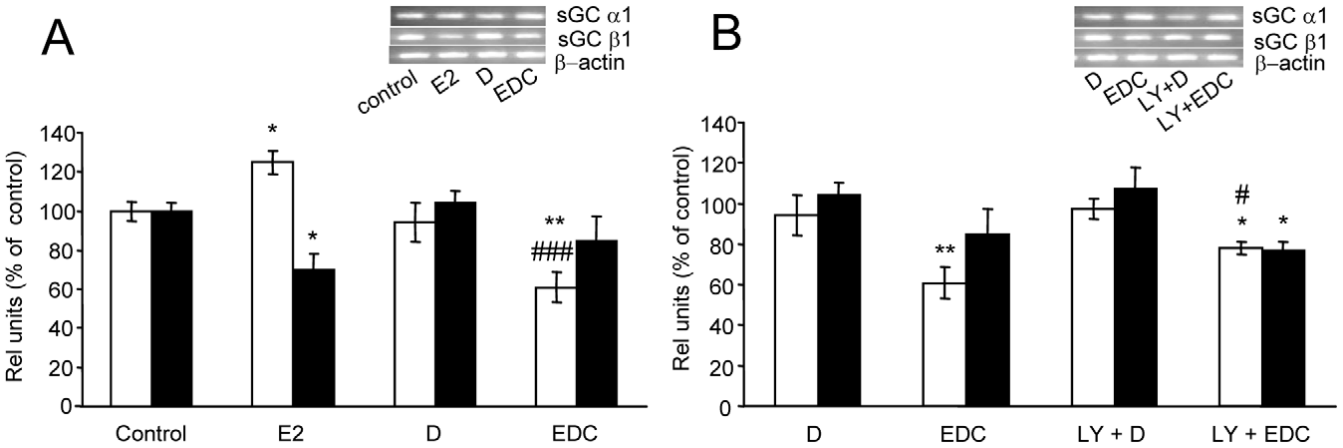
E2 decreased sGC α1 mRNA expression in a PI3k-dependent pathway involving non-nuclear ER but does not modify sGC β1 after 3 h of incubation. Pituitary cells in culture were incubated with vehicle (control) or 1 nM estrogen dendrimer conjugate (EDC), unable to trespass cellular membrane for 3 h with or without 50 µM LY294002 (LY), a PI3K inhibitor, 30 min before treatment. mRNA expression was evaluated by PCR. (**A, B**) *Top*, a representative PCRs. *Bottom*, Corresponding average densitometric values. Bars represent mean ± SE of relative units corresponding to α1 (open bars) and β1 (black bars) mRNA densitometric values normalized to β-actin, as percent of control (n = 3). ANOVA followed by Tukey's test, *P<0.05, **P<0.01 *vs*. respective controls; #*P*<0.05, ###*P*<0.001 *vs*. EDC.

**Figure 4:**
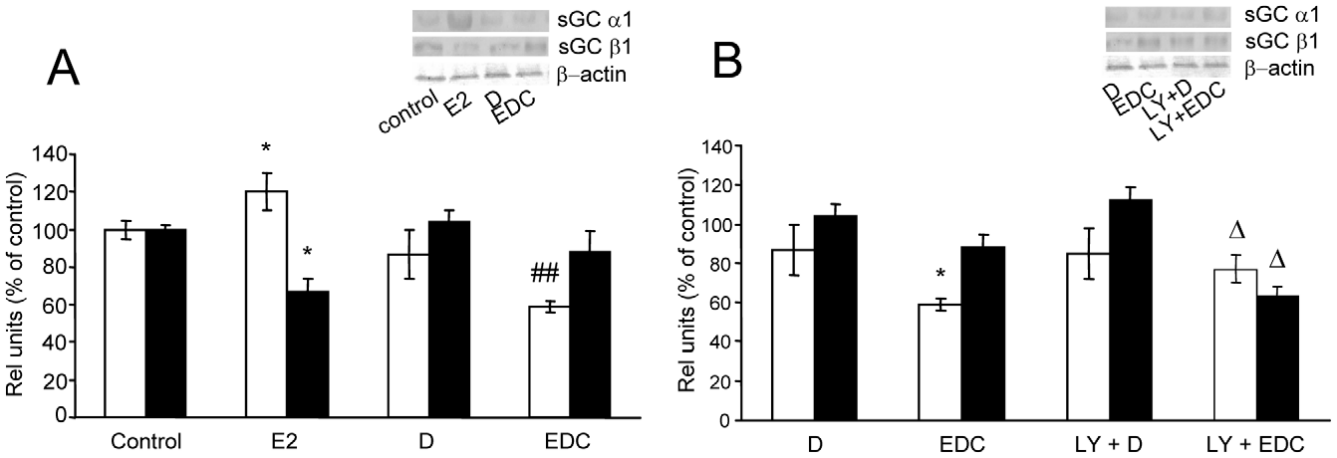
E2 decreased sGC α1 protein expression in a PI3k-dependent pathway involving non-nuclear ER but does not modify sGC β1 after 3 h of incubation. Pituitary cells in culture were incubated with vehicle (control) or 1 nM estrogen dendrimer conjugate (EDC), unable to trespass cellular membrane for 3 h with or without 50 µM LY294002 (LY), a PI3K inhibitor, 30 min before treatment. Protein expression was evaluated by western blot. (**A, B**) *Top*, representative western blots. *Bottom*, Corresponding average densitometric values. Bars represent mean ± SE of α1 (open bars) and β1 (black bars) protein densitometric values normalized to β-actin, as percent of control (n = 3). ANOVA followed by Tukey's test, *P<0.05 vs. respective controls; ΔΔ*P*<0.01 *vs*. E2; #*P*<0.05 *vs*. EDC.

Since the effects through non-nuclear ER take place at short times after E2 administration we studied the effect of E2 on subunits expression after 3 h of treatment and examined whether they are mediated through non-nuclear ER. As shown in **Figures 3A** and **4A** and in agreement with previous report [6], free E2 increased α1 and decreased β1 mRNA and protein expression. Unexpectedly, at this time, EDC significantly decreased α1 mRNA and protein expression (**Figure 3A** and **4A**, respectively), an effect opposite to that seen after 3 h and 6 h of free E2 incubation (**Figure 2**). Conversely, EDC did not significantly modify β1 mRNA or protein expression after 3 h (**Figure 3A** and **4A**). At earlier times of treatment (1 h and 2 h) neither free E2 nor EDC were able to modify α1 or β1 mRNA expression (data not shown).

These results show that E2 affects sGC α1 and β1 expression through both, non-nuclear and nuclear ER pathways in anterior pituitary cells and displays different actions depending on the pathway involved.

### Mechanisms involved in E2 effects mediated by non-nuclear ER

One of the first steps starting upon union of ligand to extranuclear ER is the activation of phosphoinositide 3-kinase (PI3K) [26]. To further confirm that E2 is acting through non-nuclear ER, cells were co-incubated with EDC and 50 µM LY294002 (LY), a specific inhibitor of PI3K. Results show that LY had no effect by itself on sGC subunits expression. LY completely blocked EDC actions on sGC α1 subunit expression at both, mRNA and protein levels, while did not modify sGC β1 subunit expression as compared with EDC effects (**Figures 3B** and **4B**).

Altogether, these results suggest that E2 modifies sGC expression through both nuclear and non-nuclear ER pathways. Activation of non-nuclear ER-initiated, PI3k-dependent pathway transiently decreases sGC α1 subunit. This effect is completely opposite when E2 acts through nuclear ER signaling pathway. On the other hand, E2 only decreases sGC β1 subunit through nuclear ER signaling pathway. Considering that after 6 h, nuclear ER signaling pathway is clearly predominant, therefore this time was chosen to study the mechanisms involved in E2 effects on sGC subunits.

### Mechanisms involved in E2 effects mediated by nuclear ER: E2 actions on sGC subunits expression are dependent on *de novo* transcription

**Figure 5:**
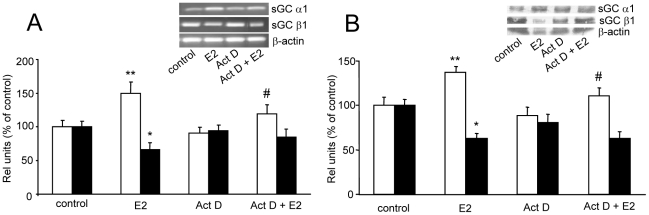
E2 actions on sGC subunits expression depended on *de novo* transcription. Pituitary cells in culture were incubated with vehicle (control) or 1 nM E2 for 6 h with or without 2 µM actinomycin D (Act D), a transcription inhibitor, 30 min before treatment. (A), mRNA expression was evaluated by PCR. *Top*, a representative PCR. *Bottom*, Average densitometric values. Bars represent mean ± SE of relative units corresponding to α1 (open bars) and β1 (black bars) mRNA densitometric values normalized to β-actin, as percent of control (n = 3). (B), protein expression was evalulated by western blot. *Top*, a representative western blot. *Bottom*, Average densitometric values. Bars represent mean ± SE of relative units corresponding to α1 (open bars) and β1 (black bars) protein densitometric values normalized to β-actin, as percent of control (n = 3). ANOVA followed by Tukey's test, *P<0.05, **P<0.01 *vs*. respective controls; Δ*P*<0.05 *vs*. E2.

Considering that α1 mRNA expression is significantly up-regulated by E2 after 6 h, we investigate if the *de novo* transcription of α1 is involved in such effect. To this end, pituitary cell cultures were incubated with 2 µM actinomycin D (Act D), a transcription inhibitor, added 30 min before 1 nM E2 incubation. Results show that Act D had no effect *per se* on α1 and β1 mRNA expression but was able to partially abolish E2-induced α1 increase (**Figure 5A**). It is possible that E2 effects on α1 subunit exceed the inhibitory activity of Act D at the dose studied. With regard to β1 subunit, Act D did not block E2-induced β1 down-regulation. A similar pattern was observed at the protein level by western blot (**Figure 5B**). These results suggest that E2 effects on α1 expression are dependent on *de novo* transcription.

### E2 effects on sGC mRNA subunits expression are fully dependent on *de novo* translation

**Figure 6:**
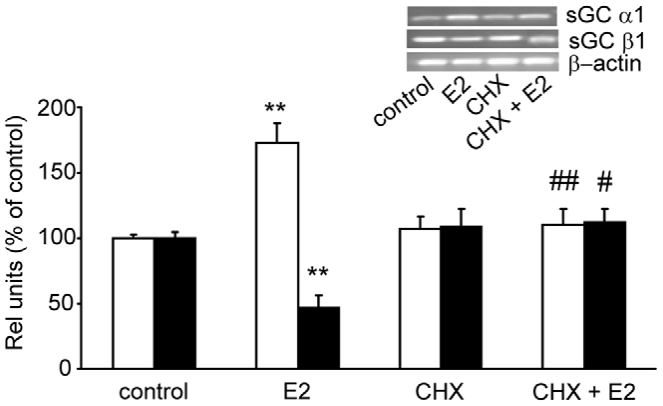
E2 actions on sGC α1 and β1 mRNAs depended on *de novo* translation. Pituitary cells in culture were incubated for 8 h with vehicle (control) or 1 nM E2 with or without 10 µg/mL cycloheximide (CHX), a translation inhibitor. mRNA expression was evaluated by semiquantitative PCR. *Top*, A representative PCR. *Bottom*, average densitomentric values. Bars represent mean ± SE of relative units corresponding to densitometric values of α1 (open bars) and β1 (black bars) normalized to β-actin, expressed as percent of control (n = 3). ANOVA followed by Tukey's test, **P<0.01 *vs*. respective controls; Δ*P*<0.05, ΔΔ*P*<0.01 *vs*. E2.

E2 genomic actions typically involve both *de novo* transcription and translation of certain factors. E2 effects on sGC mRNA transcription could be mediated by different transcriptional factors. To know whether E2 effects on sGC α1 and β1 mRNA levels depend on *de novo* translation, anterior pituitary cell cultures were preincubated with 10 µg/mL cycloheximide (CHX), a protein synthesis inhibitor, 30 min before 1 nM E2 treatment. After 6 h, total RNA was isolated and α1 and β1 sGC mRNA expression was evaluated by PCR. CHX by itself had no effect on sGC mRNAs expression, but, when co-administered with E2, was able to fully abolish E2 effects on α1 and β1 mRNA levels (**Figure 6**). This result shows that protein factors induced by E2 regulate mRNA levels of sGC subunits.

### E2 promotes changes in the expression of factors that regulates mRNA stabilization

It has been previously reported that both, α1 and β1 mRNAs are able to bind AREs binding proteins that regulate their half-lives in aortic tissue. One of the main AREs binding proteins is HuR which has been shown to be involved in the regulation of sGC mRNAs stabilization in other tissues [18]. AREs sequences are also binding targets of other regulatory proteins, such as AUF1. This protein is one of the main competitors with HuR for binding to target mRNA elements. Four isoform proteins (p45, p42, p40, and p37) of AUF1 are generated by alternative splicing and upon binding to AREs, both AUF1p37 and AUF1p42 promote mRNAs destabilization, directing them to exosomes for degradation [27]. Unlike AUF1p37, AUF1p42 includes exon 7. On the other hand, AUF1p40 and AUF1p45 include an mRNA stabilization domain in exon 2 [28]. However, it remains unknown if AUF1 and HuR are expressed in pituitary and if there is a functional relationship with E2 in anterior pituitary gland.

**Figure 7:**
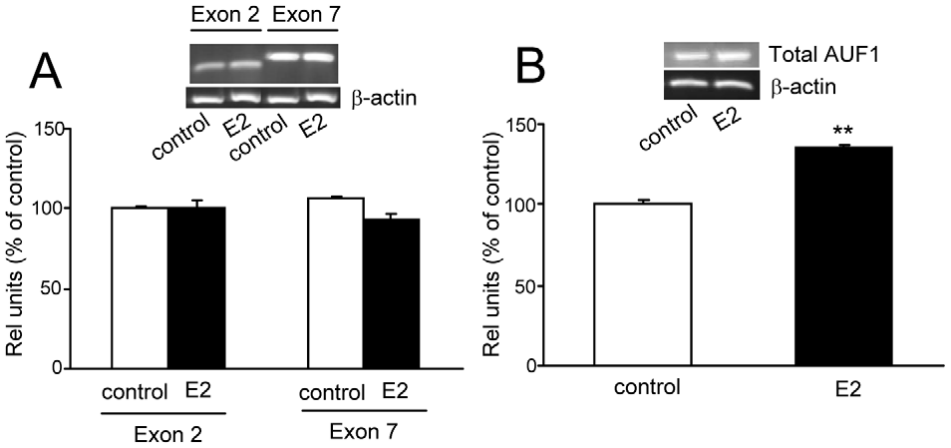
E2 treatment increases AUF1p37 mRNA expression in anterior pituitary gland. Pituitary cells in culture were incubated for 6 h with vehicle (control) or with 1 nM E2. Exon 2 and exon 7 from AUF1 (A) or expression of a conservated domain of AUF1 (B) were evaluated by semi-quantitative PCR. *Top*, a representative PCR. *Bottom*, average densitometric values. Bars represent mean ± SE of relative units corresponding to densitometric values of exon 2 and exon 7 (A) and AUF1 conservated domain (B) normalized to β-actin, expressed as percent of control (n = 3). Student's ‘*t*’ test, **P<0.01 *vs*. control.

We first demonstrated, by PCR using specific primers, that HuR (data not shown) and all exon 2- and exon 7-containing AUF1 species (p45, p42 and p40) were constitutively expressed in pituitary (**Figure 7A**). Then we investigate whether HuR and AUF1 species are involved in E2-sGC mRNA regulation in anterior pituitary. We show that 6 h of E2 treatment significantly down-regulated HuR mRNA levels (Relative units (IOD) as % of control; control: 100±8, E2: 43±5**, **P<0.01, Student's *t* test) whereas the expression of AUF1 species (p45, p42 and p40) remained unchanged (**Figure 7A**). However, E2 increased AUF1 expression detected by primers directed to a conserved domain among the four AUF1 isoforms (**Figure 7B**). Taking into account that p45, p42 and p40 expression did not modify after E2 treatment, it can be stated that E2 induced an augment of p37 isoform.

The effects of nuclear and non-nuclear E2 pathways on HuR and AUF1 expression were also addressed at earlier times. After 3 h of treatment, neither free E2 nor EDC were able to significantly modify HuR or AUF1p37 mRNA levels (data not shown).

Altogether, these results suggest that E2, by down-regulating HuR mRNA levels and increasing p37 isoform, promotes an environment of AREs-containing mRNAs destabilization in anterior pituitary gland.

## Discussion

Both subunits of sGC, α1 and β1, are constitutively expressed in anterior pituitary gland. sGC is found in all hormone-producing cells, while follicle-stellate cells are the only cell type lacking sGC (unpublished data). The wide distribution of α1 and β1 in the gland suggests the key role of sGC in the NO pathway in anterior pituitary.

In this work we demonstrate that E2 modifies sGC α1 and β1 subunits expression in anterior pituitary gland through classical and non classical pathway. Non-nuclear ER-dependent E2 actions on both subunits are different to that mediated by nuclear ER. E2 exerts an opposite effect on α1 depending on the activated pathway. E2 through non-nuclear ER, PI3k-dependent pathway transiently decreases α1 subunit expression while through nuclear ER pathway augments α1 levels. On the other hand, E2 only modifies β1 subunit expression through nuclear ER. This evidence could reveal a new level of regulation in NO/sGC pathway which role in sGC regulation opens a new field of study with interesting questions to be addressed.

It has been previously shown that E2 exerts a short- and long-term decrease of sGC activity in anterior pituitary gland [5], [29]. The non-nuclear actions of E2 could be of major importance in the regulation of sGC activity at very short times, at the level of membrane microdomains where sGC can also be found [30]. Thus E2 by decreasing α1 expression -the limiting subunit of sGC- could be able to reduce cGMP production. Therefore, α1 levels are first down-regulated probably due to the mRNA destabilization environment promoted by E2 [31] but augmented later through E2 stimulatory effect on α1 transcription. Even though our results have showed that at short times the mRNA levels of HuR and AUF1 remain unchanged, it cannot be ruled out that E2 through non-nuclear pathway could affect HuR and AUF1 activity and/or availability, which seems more likely as a rapid E2 effect. Besides, it is also possible that other factors could be involved in E2 non-nuclear actions. This topic opens a new field which is currently under investigation.

On the other hand, at longer times E2 seems to down-regulate sGC activity mainly by decreasing β1 expression, given that α1 increased levels are probably playing other role on cell physiology. In that way, we have previously shown that α1 expression positively correlated with proliferative stages of estrous cycle [6] and its expression was found significantly augmented in pituitaries of chronic-estrogenized female rats, although sGC activity is diminished in both cases (unpublished data).

The E2 actions through nuclear and non-nuclear E2 pathways could be of major relevance, not only on sGC regulation, but also for many factors regulated by E2 in this gland. Up to date, the paradoxical response to E2 depending on the pathway involved was demonstrated only in bone [32]. Here, we provide the first evidence that E2 nuclear and non-nuclear pathways trigger different and opposite actions in anterior pituitary gland. We demonstrate that E2-membrane effects on sGC expression that take place at short times were transient and no longer evident after 6 h.

We also studied the mechanisms by which E2 differentially modifies α1 and β1 expression in anterior pituitary. We have demonstrated that, after 6 h treatment, E2 actions on sGC expression are mediated through activation of nuclear ER. Thus, the regulation of sGC expression could happen mainly at transcriptional/post-transcriptional levels. Our results show that E2 regulation of α1 depends on both, *de novo* transcription and translation while β1 expression depends only on *de novo* translation. Once again, these findings support the hypothesis of independent regulation of sGC subunits.

E2 can also regulate many genes post-transcriptionally by affecting mRNA stability. As previously mentioned, adenine-uracil rich elements (AREs) are involved in rapid mRNA degradation and are binding targets of several proteins thus constituting important elements for the regulation of genetic expression in vertebrates. HuR protein binds to AREs [12] and protects mRNA body from degradation. It has been showed that, at the post-transcriptional level, HuR stabilizes α1 as well as β1 mRNAs and actively protects them against degradation [18], [33]. Here we demonstrated that HuR is expressed normally in anterior pituitary gland and is down-regulated after E2 acute treatment. On the other hand, AUF1 protein is one of the main factors regulating AREs-containing mRNA half-lives. Our results indicate that all AUF1 isoforms are normally expressed in anterior pituitary and that after E2 treatment only destabilizing isoform AUF1p37 is augmented. This observation is in agreement with previous evidence showing that E2 treatment was able to decrease the expression of several proteins involved in mRNA stabilization in anterior pituitary gland [30].

Both, α1 and β1 mRNAs yields AREs elements; however, they are differentially affected by E2. Our results underline the hypotheses that E2 affects β1 mRNA levels by stimulating the expression of proteins involved in mRNA destabilization processes. The decrease of β1 levels after E2 treatment could be a consequence of E2 actions on mRNA stabilization proteins. In that way, α1 mRNA can also be target of degradation but, as transcription is over enhanced, the global balance results in higher α1 mRNA expression. Besides, it cannot be excluded that α1 mRNA could be protected from degradation through binding of other factors or that other factors could be affecting α1 expression. Genes of α1 and β1 subunits lack of estrogen response elements (ERE) in their sequences, but they include consensus sites for binding of Sp-1, c-Myb and NFκ-B, among others, in their promoter regions [34]. These factors are regulated by E2 [35]-[37] so it could be possible that E2 regulates sGC subunit expression through modulation of these proteins. Although sGC α1 and β1 promoter regions have many binding sites for different factors in common, they yield independent activities, which together with their separated location in the chromosome, support the hypothesis of independent regulation of both genes [38]. These facts could explain the differential response of both genes to E2 action [39].

Evidence provided by us, together with reports of temporal unbalance in α1 and β1 expression [8] and participation of α1 and β1 individually in cell cycle regulation [34], [35] support the hypotheses that the imbalance of sGC subunits expression exceed the solely regulation of enzimatic sGC activity. It was previously reported that α1 acts in prostate cancer via a novel pathway that does not depend on β1 and that its expression correlates with advanced prostate cancer. Thus, α1 has been proposed as an important mediator of the procarcinogen effects of androgens [34]. On the other hand, β1 was found associated with chromosomes during mitosis in neural cells negatively regulating cell cycle progression [35]. Thus, besides comprising sGC enzyme, α1 and β1 independently appear as multifunctional proteins with key roles in cell processes.

In summary, this work provides the first approach that both sGC subunits are independently regulated and that E2 through different mechanisms affects α1 and β1 sGC expression, which strongly remarks the individual importance of sGC in processes not related to cGMP synthesis in pituitary. This contributes to the understanding of α1 and β1 roles in pituitary physiology which will advance in future investigations.

## Materials and Methods

### Ethics Statement

All experimental procedures were approved by the Committee on Ethics of the School of Medicine (University of Buenos Aires, Resolution No. 1889/06) and were carried out in compliance with the guidelines of the NIH Guide for the Care and Use of Laboratory Animals.

### Materials

Go Taq DNA polymerase, random hexamers and dNTPs were provided by Promega (Madison, WI). TRIzol and molecular biology reagents were from Invitrogen (Carlsbad, CA). Media and reagents for cell culture were purchased from Gibco (Rockville, MD, USA), except for the fetal bovine serum that was obtained from GBO (Buenos Aires, Argentina). 17β-estradiol (E2), β-estradiol 6-(O-carboxymethyl)oxime:BSA (E2-BSA), LY294002 and all other reagents and antibodies were obtained from Sigma (St. Louis, MO). Estrogen dendrimer conjugate (EDC) was kindly gifted by Dr. John A. Katzenellenbogen (University of Illinois at Urbana-Champaign).

### Preparation of E2-BSA free of E2

400 µL of E2-BSA (10^−5^ M estrogen dissolved in 50 mM Tris, pH 8.5) was added to a centrifugal filter unit with a MW cut-off of 3,000 (Millipore) and centrifuged at 14,000 × g until 50 µL of retentate remained. The retentate was washed 3 times with 350 µL of buffer, recovered and volume adjusted to 400 µL.

### Animals and treatments

Adult female Wistar rats (180–200 g) were used at random stages of estrous cycle. Animals were kept with controlled conditions of light (12∶12 h light/dark cycle) and temperature (21–24 C). Food and water were supplied *ad libitum*.

### Cell culture

Anterior pituitary glands (n = 10) were removed within minutes after decapitation. In females, anterior pituitary cell population is composed by 52% lactotrophs, 20% somatotrophs, 10% gonadotrophs, 3% corticotrophs, 2% tirotrophs and 13% immunonegative cells [40]. Cells were obtained by enzymatic (trypsin/DNAse) and mechanical dispersion (extrusion through a Pasteur pipette) as previously described [5]. Cell viability was assessed by the trypan blue exclusion method. In all cases, viability was higher than 90%. Dispersed cells were seeded onto 24-well tissue culture plates (1.10^6^ cells/well) and stabilized for 48 h (37 C, 5% CO_2_ in air) in phenol red-free Dulbecco's modified Eagle's medium (DMEM) supplemented with 10% charcoal stripped fetal bovine serum (CSFBS), 10 µL/mL MEM amino acids, 2 mM glutamine, 5.6 µg/mL amphotericin B and 25 µg/mL gentamicin (DMEM-S-10% CSFBS).

### Cell treatment

After the stabilization period (48 h), medium was changed for fresh medium and cells were incubated during 3 or 6 h (37 C, 5% CO_2_ in air) with 1 nM E2 or EDC. Control treatment was carried out with complete culture media containing vehicle at the same final concentration used for E2 (diluted in 1 nM ethanol) or EDC treatments (diluted in 1 nM methanol). Different drugs were pre-incubated for 30 min before E2 treatment, to avoid interactions between E2 and drug kinetics. After treatment, RNA isolation or protein extraction of each condition was carried out.

### RNA isolation

177 µL of TRIzol reagent was added to each well. After isolation, total RNA from tissues was spectrophotometrically quantified at 260 nm. RNA integrity was checked in formaldehyde/formamide gel electrophoresis.

### RT and PCR reactions

First strand cDNA was synthesized with Moloney murine leukemia virus (M-MLV) reverse transcriptase in RT buffer containing 5.5 mM MgCl_2_, 0.5 mM dNTP, 2.5 µM random hexamers, and 3.125 U/µL M-MLV reverse transcriptase. Reactions were done in a final volume of 12 µL containing 1 µg RNA. The reverse transcription reaction was run at 37 C for 50 min and reverse transcriptase was inactivated by heating the samples at 70 C for 15 min before the PCR reactions. To check for genomic contamination, the same procedure was performed on samples in a reaction solution lacking reverse transcriptase.

Specific primers for both subunits of sGC were designed from published sequences [6] with Oligo Perfect designer software (Invitrogen) and are detailed in **Table 1**. The amplified products spanned from nucleotide position base 1971–2054 in the C-terminal region of sGC α1 (GenBank accession number NM_017090); from 714–823 in the N-terminal region of sGC β1 (M22562); from 741–955 in the N-terminal region of HuR (NM_001108848); from 649–674 in the middle region of p45AUF1 (AB_046615), from 503–574 in the A cassette (N-terminal region) of p45AUF1 and from 1077–1179 in the B cassette (C-terminal region) of p45AUF1. β-actin (NM_031144) was used as an endogenous control. Actin primers were designed in order to detect amplification of DNA contamination. Then, samples were thermocycled for PCR amplification (Mastercycler, Eppendorf, Hamburg, Germany). The reaction mixture contained GoTaq PCR buffer, 1.5 mM MgCl_2_, 200 µM of each dNTP, 0.625 U GoTaq polymerase and 300 nM of each primer. We utilized RT-PCR methods to determine relative changes in mRNA expression. Reactions were subjected to a varying number (n = 16–40) of cycles of PCR amplification (melting phase 94 C for 30 sec, annealing 55 C for 30 sec and extension 72 C for 1 min) to find out the optimum cycle number within the linear range for PCR amplification. Amplified products collected at various cycles were analyzed by electrophoresis in 1.5% agarose-ethidium bromide gels, and the optimum cycle number resulted to be 24 cycles for β-actin, 28 cycles for sGC α1 and sGC β1 and 40 cycles for HuR and AUF1.

**Table 1:** Primers used for semi-quantitative RT-PCR assays.

Gene		Primers	Product size (pb)
sGC α1	Forward	5′-ACACAATATGCATCTCCGATGG-3′	83
	Reverse	5′-GCTCTCTATACTCGCTTTGACCAA-3′	
sGC β1	Forward	5′-CCCGTGGAAACTGATGTCAA-3′	109
	Reverse	5′-CGGGACCTAGTAGTCACGCA-3′	
HuR	Forward	5′-TCGCAGCTGTACCACTCGCC-3′	214
	Reverse	5′-CCAAACATCTGCCAGAGGATC-3′	
AUF1	Forward	5′-GTAGACTGCACTCTGAAGTTAGATCC-3′	450
	Reverse	5′-CTCCTCTAGATCCCCACTGCTG-3′	
AUF1 Exon 2	Forward	5′-AGGATGAAGGCCATTCAAAC-3′	71
	Reverse	5′- TTTTCCATTCTTCCCGCTG-3′	
AUF1 Exon 7	Forward	5′-CCCCAGTCAAAACTGGAAC-3′	102
	Reverse	5′-AGTCATATCCTCCATAACCACC-3′	
β-actin	Forward	5′-ACCACAGCTGAGAGGGAAATCG-3′	276
	Reverse	5′-AGAGGTCTTTACGGATGTCAACG-3′	

### Analysis of semi-quantitative PCR data

The intensity of PCR products was determined by digital image analysis using the Gel Pro Analyzer (Media Cybernetics, LP, Silver Spring, MD) software for Windows. To allow statistical comparison of results from different experiments, sGC subunits levels were normalized to the value of the β-actin amplified band in each lane.

### Preparation of cell homogenates for immunoblot analysis

Anterior pituitary cells in culture were tripsinized and sonicated in lysis buffer containing 10 mM HEPES pH 7.4, 150 mM NaCl, 10 mM EDTA, 100 µM leupeptin, 350 µM pepstatin, 0.5 mM PMSF and 0.2 mM DTT. Homogenates were centrifuged for 20 min at 10,000 x *g* (4 C) and the post-mitochondrial fraction was used in the immunoblot analysis.

### Protein measurement

Protein content of the supernatants was measured by Bradford reagent, using bovine serum-albumin as standard.

### Immunoblot analysis

Fifty micrograms of total protein from each sample was boiled for 5 min in Laemmli sample buffer and fractioned on 10% SDS-PAGE. Resolved proteins were transferred to polyvinylidene difluoride membranes and blocked for 2 days at 4 C in blocking buffer (TBS-0.05% Tween 20, 6% nonfat dry milk). Then, membranes were co-incubated overnight at 4 C with rabbit antisera anti-sGC α1 (1∶1750) or β1 (1∶700) subunits together with anti-actin (1∶1000) in blocking buffer. Blots were washed and incubated for 1 h at room temperature with horseradish-peroxidase conjugated goat antirabbit IgG (1∶2000), followed by detection of immunoreactivity with diaminobenzidine solution containing 0.01% hydrogen peroxide.

### Analysis of immunoblot data

The intensity of immunoblot signals was determined by digital image analysis using Gel Pro Analyzer (Media Cybernetics, L.P.) software for Windows. To allow statistical comparison of results from different blots, levels were normalized to the value of the actin immunoreactive band in each lane.

### Statistical analysis

Results are expressed as mean ± SE and evaluated by one-way ANOVA followed by Tukey's or Student's *t* test, depending on the experimental design. Differences between groups were considered significant if *P*<0.05. Results were confirmed by at least three independent experiments.
